# Mentorship as a secure base: an attachment and mentalizing perspective on academic development

**DOI:** 10.3389/fpsyg.2026.1857882

**Published:** 2026-09-04

**Authors:** Karen Yirmiya, Peter Fonagy

**Affiliations:** 1 Department of Psychology, Ben-Gurion University, Be'er Sheva, Israel; 2 Research Department of Clinical, Educational and Health Psychology, University College, London, United Kingdom

**Keywords:** academic development, attachment, epistemic trust, higher education, mentalizing, mentorship

Early academic careers unfold under substantial psychological strain, and the transition from student to researcher often brings sustained uncertainty, self-doubt, and intense social evaluation. Mentorship plays a central, yet undertheorized, role in shaping scientific development, mental health, and resilience (Bergvall et al., 2025; Nicholls et al., 2022). Emerging work indicates that relational processes within advisory relationships may be as consequential as cognitive or technical skill (Liu et al., 2024; Radha Krishna et al., 2019). Prior work, including Goshen and colleagues (2022), highlights mentors who support scientific standards alongside broader professional growth (Goshen et al., 2022). Here we foreground a clinical-developmental lens on the relational conditions that enable people to think, learn, and grow. We outline the advisor's role as a mentalizing agent and, at best, a secure base within an attachment relationship, and propose that advisors' ostensive, marked, and contingent behaviors foster epistemic trust (ET), downregulate threat, and enable exploration and academic risk-taking-processes essential for creativity and wellbeing. Cultivated deliberately, these dynamics may protect against anxiety, depression, and burnout in contemporary academic life (Bergvall et al., 2025).

## Attachment foundations of mentorship

Attachment is a biologically rooted system across the lifespan. In infancy, caregivers function both as safe haven, providing protection, soothing, and emotion regulation during distress, and as secure base from which the child explores with curiosity and confidence (Bowlby, 2005). Through attuned, contingent responsiveness, children build internal working models-representations of self and other that organize expectations about whether feelings make sense, whether others will understand them, and whether support will be reliably available. From a mentalizing perspective, ostensive cues, marked mirroring, and contingency render the child's mind “*graspable”* to itself. These models continue to guide adult relationships, including mentoring, and can serve analogous regulatory and exploratory functions in times of need. Repeated empathic validation, affective attunement, and reflective commentary, verbal and nonverbal, are the vehicles of mentalizing that make one's own and others' thoughts and feelings coherent and thinkable.

## Epistemic trust as a learning mechanism

These developmental processes matter in academia. Secure attachment predicts long-term outcomes, such as stronger self-efficacy, academic engagement, and academic success (Ramsdal et al., 2015). Studies of academic resilience show that supervisors' and teachers' relational qualities predict performance and wellbeing (Cai and Meng, 2025; Eby et al., 2008). Across disciplines, mentoring that builds trust, safe learning environments, and psychological safety is associated with perceived innovation, job engagement, and complex skill acquisition (Moore and Wang, 2017; Radha Krishna et al., 2019). In medical education, timely feedback, developmental tailoring, and spaces in which students can safely make mistakes are strong predictors of psychological safety (Thyness et al., 2023). Mentoring is distinct from teaching by its relational, personalized, longitudinal, developmentally scaffolded support (Radha Krishna et al., 2019). Together, these literatures suggest that secure-attachment-like qualities can revise or consolidate internal models that underwrite “*academic selves”*. Mechanistically, when advisors deploy ostensive cues, attention, contingent responsiveness, affective marking, and explicit perspective-taking, mentees experience reduced social threat and increased ET, i.e., openness to receiving knowledge from another based on a felt sense of benevolence, attunement, and good intent (Fonagy and Allison, 2014). ET, in turn, supports exploratory behaviors, including risk-taking, initiative, and persistence after failure, which accumulate as productivity, wellbeing, and retention.

Good advisership involves discovering and making room for the mentee's mind. Comparable attachment functions are recruited in response to normative academic stressors, such as grant triage, difficult revise-and-resubmit decisions, and disputes about authorship and credit. Mentorship thus functions as an adult attachment relationship in which psychological safety enables exploration, creativity, and scientific risk-taking. Advisors also serve a safe-haven role by tracking and naming the mentee's fluctuating affective and epistemic stance under evaluation, fear of failure, impostor feelings, disappointment, hopelessness, and the discouragement that accompanies rejection. By marking these states as internal experiences rather than verdicts on ability (e.g., “*this is your fear, not the state of reality”*), advisors help preserve a reflective stance and prevent hyperactivation (over-seeking reassurance) or deactivation (withdrawal and avoidance). Held consistently and without judgment, such representations allow mentees to consolidate an “*academic self”*. Of course, advisorship can also be misattuned or destructive. Psychological threat and chronic evaluation anxiety are well documented to undermine learning and performance (Radha Krishna et al., 2019). When mentees' emotional states are dismissed, mocked, or absorbed without appropriate marking, threat is amplified and ET erodes, weakening both exploration and resilience.

Today, with AI offering immediate access to knowledge and quasi-emotional support, the question of what remains uniquely human in relationships is sharper. As a boundary case, we ask whether AI can instantiate the attachment functions at stake: safe haven, secure base, and mismatch-repair. According to mentalizing theory, human relationships are defined by genuine affective commitment and shared intentionality, expressed through subtle, embodied attunement that allows two minds to resonate (Fonagy and Allison, 2014). These capacities, the warmth behind an ostensive cue, the moral agency that underlies trust, and the willingness to invest effort and care, remain human. Emerging evidence suggests that people can form attachment-like bonds with AI systems, best characterized as “*as-if”* attachments in current LLM- and rule-base systems, with developmental consequences that differ from relationships with fully intentional others (Yirmiya and Fonagy, 2025).

As AI systems are increasingly embedded in academic research and everyday scholarly life, supporting writing, analysis, feedback, and decision-making, they are beginning to occupy some roles that intersect with mentoring and guidance. While AI can simulate aspects of cognitive empathy, current systems do not yet demonstrate reciprocal, uncertainty-laden mentalizing in moment-to-moment interaction with a specific mentee. ET depends on interactive contingency, especially mismatch-and-repair processes that stabilize attachment bonds, which current systems do not implement in a truly bidirectional, mentation-sensitive way (Miller-Bottome et al., 2019). Because AI lacks intentionality, emotional investment, and collaborative repair, its supportive functions cannot substitute for the human abilities required in advisership. Mentalizing, the foundation of ET, cannot at present be fully instantiated by synthetic systems, nor can they truly “hold someone in mind”.

## Implications, vulnerabilities, and limitations

The following implications describe the relational behaviors that operationalize the proposed mechanism linking mentorship to epistemic trust, exploratory learning, and mental health outcomes. A good mentor-mentee relationship cultivates ET through ordinary but potent moves: honesty, transparency, and light humor; an active search for synchrony with pace and expectations; and an internalized egalitarian, not-knowing stance that disrupts unidirectionality of knowledge flow and recognizes the mentee as the primary navigator. Understanding is built jointly rather than delivered; the advisor offers tools (methods, heuristics, networks) as ostensive signals that the mentee's perspective is the organizing frame, enabling the mentee to shape their academic narrative, rather than having problems solved for them. Power differentials are softened by judicious self-disclosure, openness to a “*We-mode”* of shared intent, and consistent enabling of self-control, decision-making, and agency, while acknowledging the unavoidable assessor role. Advice is collaborative, with co-constructed conclusions. Trust in predictable supervisory routines and in the research program are made explicit, providing predictable yet flexible structure; contextual cues, including in remote meetings, sustain connection. Advisors also legitimize change arising outside supervision: self-initiated efforts, encounters beyond the lab, family and social supports, community ties, and fortuitous opportunities—and link these to progress. ET is thus an ongoing experience rather than a destination: ET increases mentalizing; mentalizing supports exploration; and exploration yields mastery experiences that reinforce ET.

Relationships vary in rhythm and boundaries, the extent of personal conversation, the pace of interaction, and the balance between closeness and autonomy, under conditions of chronic evaluative threat. The core remains an advisor's openness to needs, interest in thoughts and feelings, and willingness to engage sincerely at moments of difficulty. Attachment dynamics are complex: too much closeness can inhibit mentalizing; too much distance impedes recognition. In academia, the advisor's simultaneous role as evaluator and gatekeeper heightens this tension, requiring secure-base functions under chronic evaluative threat. Clear expectations and criteria, transparent decision-making, and collaborative goal-setting and authorship can offset this pressure and support psychological safety. For first-generation and minoritized scholars, who may enter with lower baseline ET toward institutions, explicitness and cultural attunement matter even more; mentors who display cultural awareness consistently provide more supportive, higher-quality mentoring regardless of demographic similarity (Tuma and Dolan, 2024). Baseline ET may be further depressed by structural conditions that fall outside the mentor-mentee dyad, including visa and immigration precarity, disproportionate caregiving loads, and unmet disability or neurodivergent accommodation needs. In such contexts, secure-base functions cannot rest solely on an individual advisor but may need to be scaffolded through multi-mentor models, lab-level climate, and institutional practices that distribute recognition, protection, and epistemic support. While the dyad remains central, epistemic trust can be sustained or eroded by the wider relational ecology in which mentoring is embedded.

This framework lends itself to empirical tests. If the proposed mechanism is correct, mentors' ostensive signaling during supervision should predict mentees' ET (e.g., ETMCQ); ET should mediate associations between supervisory alliance ratings and exploratory outputs (e.g., proposing novel ideas, submission attempts); and the frequency and quality of rupture-repair conversations should moderate persistence after rejection or critique. Advisership is shaped by a familiar tension: good advisors mentalize their students: staying close enough to guide yet far enough to allow autonomy; protecting when needed yet stepping back so mentees can discover their own capacities. The advisor–advisee relationship is a primary context in which academic minds become capable of exploration. Becoming an independent researcher is rarely straightforward, but when an advisor is also a mentor, and when that mentor becomes a secure attachment figure, the journey becomes not only more manageable but also developmentally consequential for scholarly identity and wellbeing.
